# Idiopathic Intracranial Hypertension Secondary to Chronic Inflammatory Demyelinating Polyradiculoneuropathy

**DOI:** 10.7759/cureus.43648

**Published:** 2023-08-17

**Authors:** Sedat Gül, Irine Siraj, Jenny A Meyer

**Affiliations:** 1 Neurology, State University of New York Upstate Medical University, Syracuse, USA; 2 Neurology, Cayuga Health, Ithaca, USA

**Keywords:** acetazolamide, hydrocephalus, idiopathic intracranial hypertension, papilledema, chronic inflammatory demyelinating polyradiculoneuropathy

## Abstract

Chronic inflammatory demyelinating polyradiculoneuropathy (CIDP) is the most common immune-mediated inflammatory polyneuropathy, defined as progressive or relapsing symptoms for over two months with pathological or electrophysiological evidence of peripheral nerve demyelination. Papilledema is optic nerve head edema secondary to increased intracranial pressure or infiltrative/infectious etiologies. Regardless of the cause, visual loss is one of the feared manifestations due to optic nerve damage. We present a 50-year-old female patient with CIDP who developed papilledema that was secondary to increased intracranial pressure from high protein content in the cerebrospinal fluid (CSF) and elevated body mass index (BMI) secondary to prednisone use. Treatment with acetazolamide completely resolved the papilledema and headaches, and the patient was later maintained on mycophenolate, intravenous immunoglobulin (IVIG), rituximab, and prednisone. To the best of our knowledge, this is the first case that describes successful medical management of increased intracranial pressure in the setting of CIDP.

## Introduction

Chronic inflammatory demyelinating polyradiculoneuropathy (CIDP) is the most common immune-mediated inflammatory polyneuropathy, defined as progressive or relapsing symptoms for over two months with pathological or electrophysiological evidence of peripheral nerve demyelination [1]. The prevalence of the diseases varies between 0.8 to 8.9 per 100.000 individuals [2,3].

The typical presentation is symmetric motor weakness affecting both proximal and distal muscles, and sensory ataxia. Diffuse hyporeflexia or areflexia are common findings. The presenting symptoms and above-mentioned features may suggest acute inflammatory demyelinating polyradiculoneuropathy (AIDP) on initial presentation; however, the duration of the symptoms and recurrent nature differentiates between AIDP and CIDP [1]. Nerve conduction studies and electromyography may aid with definitive diagnosis. Cerebrospinal fluid (CSF) shows albuminocytologic dissociation with elevated protein and mild pleocytosis. Nerve biopsy is also highly specific and helps exclude other diseases [4].

CIDP is primarily a T cell-mediated disease; however, both cellular and humoral immunity contribute to the pathogenesis. There is a loss of regulation of immune responses to unknown antigens [5,6]. Steroids, intravenous immunoglobulin (IVIG), plasma exchange, and steroid-sparing agents are mostly used in treatment modalities. A small percentage of patients with CIDP may experience ocular manifestations, namely ptosis, papilledema, optic neuritis, and diplopia [7,8]. Papilledema is optic nerve head edema secondary to increased intracranial pressure or infiltrative/infectious etiologies. Regardless of the cause, visual loss is one of the feared manifestations due to optic nerve damage. Imaging techniques or fundus exams are used to detect it [9]. Idiopathic intracranial hypertension (IIH) is one of the etiologies of papilledema. Its incidence is 4.7 per 100.000 and usually presents with migraine-like headache, visual loss or disturbance, and pulsatile tinnitus. It is closely related to obesity [10].

Here, we present a case of CIDP with papilledema, which was secondary to increased intracranial pressure from high protein content in the CSF and elevated BMI secondary to prednisone use. 

## Case presentation

A 50-year-old female patient with CIDP presented to the clinic with new-onset headaches spreading down her neck and causing intermittent blurry vision in both eyes for five minutes. The headaches lasted 15 minutes at most and went away without any intervention. She experienced multiple episodes of vision loss in the left eye, correlating with the headaches. The headaches did not correlate with the worsening of the CIDP symptoms. 

Her CIDP symptoms started approximately two years before with right-sided lower motor neuron type facial paralysis, dysphagia, flaccid paralysis, horizontal diplopia with bilateral cranial nerve VI palsy, and numbness in bilateral upper and lower extremities. She was initially diagnosed with AIDP and treated with IVIG. Despite this, she had recurrent attacks and progression of the disease which led to a diagnosis of CIDP over the course of three months. She could ultimately be discharged from the hospital after initiating rituximab therapy and prednisone. She was maintained on IVIG every three weeks, mycophenolate mofetil, prednisone, and rituximab therapy. 

Her exam revealed baseline visual acuity, given the transient nature of the findings. Visual fields were full. There were no proptosis or conjunctival injections. Extraocular movements were significant for bilateral abduction deficits. Bilateral ptosis was present, more prominent on the right side. She had decreased strength in all muscle groups, more prominent in the bilateral interosseous and hip flexors. Reflexes were mute and sensation to pinprick was diminished in four extremity distals. Examination of the fundi showed a hemorrhage lateral to the optic nerve at the left eye.

**Figure 1:**
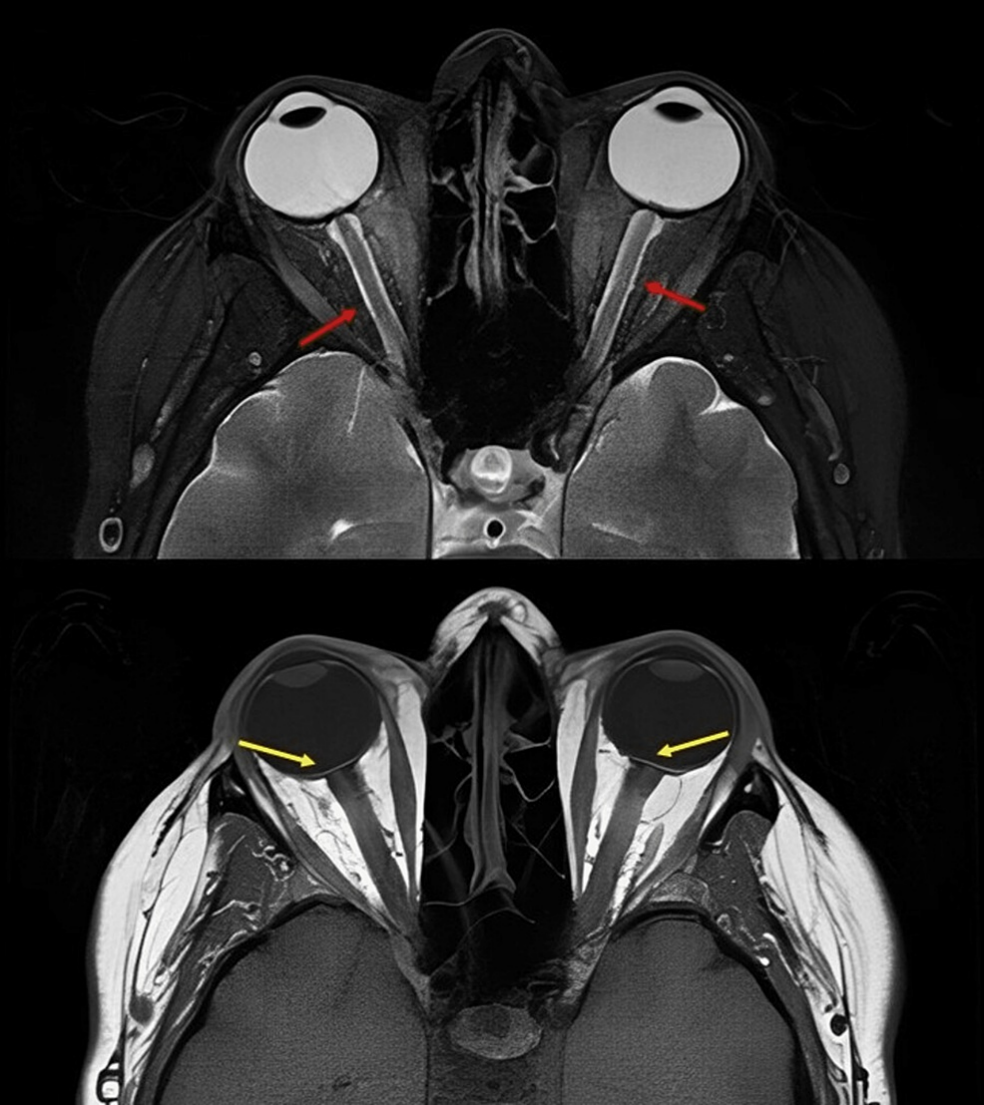
MRI of the orbit. Top image: Axial T2, prominent CSF space along the optic nerve sheath on either side (red arrows). Bottom image: Axial T1, mild flattening of the posterior sclera bilaterally (yellow arrows). These findings can be seen with increased intracranial pressure.

The patient was referred to ophthalmology and admitted to the hospital due to recurrent symptoms. Lumbar puncture was done with an opening pressure of 33 cmH2O, prootein 527 mg/dL, albumin 428 mg/dL, and total nucleated cell <3. A total of 24 ml was collected in total. The ophthalmology exam revealed papilledema and bilateral ptosis with baseline visual acuity. CSF pathogen panel, CSF lyme, CSF syphilis, and HIV screening were negative. MRI of the brain with and without contrast and MRI orbit only revealed flattening of the posterior sclera bilaterally and prominent CSF space along the optic nerve sheath with no hydrocephalus or intracranial mass (Figure 1). The patient was diagnosed with IIH.

Treatment with acetazolamide completely resolved the papilledema and headaches. Three years after the initial diagnosis of IIH, the patient is being managed with acetazolamide, mycophenolate, IVIG, rituximab, and prednisone with only mild residual sensorimotor neuropathy symptoms. 

## Discussion

CIDP is characterized by progressive or relapsing peripheral neuropathy lasting longer than eight weeks [1]. This patient was previously diagnosed with CIDP; however, she reported new symptoms including headache and blurry vision. She gained a significant amount of weight during the treatment due to steroid medications and lack of physical activity (BMI increased from 45 kg/m² to 59.7 kg/m² ). When she was first diagnosed, she had evidence of albuminocytologic dissociation (protein in CSF 386 mg/dL and total nucleated cells 3/uL ) but did not report any vision loss, only diplopia.

The new symptoms and hemorrhage in the left fundus prompted an ophthalmology referral and papilledema was detected. A more recent CSF study showed significantly increased CSF protein (527 mg/dL and total nucleated cells < 3/uL), which would potentially increase the viscosity of the CSF and cause intracranial hypertension.

Similar findings may be elicited by a space-occupying mass lesion that causes hydrocephalus. However, as stated above, brain imaging did not reveal any masses. Vascular lesions, such as amaurosis fugax may present with transient blurry vision; however, correlation with the headache and fundus findings wouldn’t be expected. As stated above, infectious, or autoimmune causes were also ruled out with CSF studies. The patient’s symptomatic response to acetazolamide and elevated opening pressure on the lumbar puncture supported the diagnosis of IIH. 

There are two previously reported cases of CIDP and IIH. A case report by Altinkaya et al. describes a similar patient but with much lower CSF protein, 119 mg/dL. This case resulted in significant improvement in headache and papilledema after prednisone treatment and repeat CSF analysis showed normalized protein [11]. Steroids are the standard of care for CIDP flare-ups [12]. However, our patient did not benefit from the steroid treatment, IVIG, mycophenolate mofetil, or rituximab for the headaches and they continued even as CIDP symptoms improved. Another case report by Fantin et al. described a patient with CIDP, with an opening pressure of 52 cmH2O and CSF protein of 745 mg/dl. This patient’s symptoms were unresponsive to maximum medical therapy and optic nerve sheath decompression was performed [13]. 

In the current case report, we described a rare case of a patient with a two-year history of CIDP who subsequently developed headaches and blurry vision, and was diagnosed with IIH possibly caused by increased CSF viscosity due to increased protein content and weight gain from prednisone. We aimed to emphasize the importance of keeping IIH in the differential for headache or vision changes in CIDP patients as a possible complication to prevent catastrophic outcomes. 

## Conclusions

CIDP usually manifests with inflammatory polyneuropathy in a relapsing pattern. However in our patient, in addition to the classic manifestations of CIDP, we detected signs of increased intracranial pressure. When investigated, the patient's CSF protein was significantly elevated and she had recent weight gain. Treatment of increased intracranial pressure improved the symptoms independent of the CIPD progression. It is important to have a comprehensive look when evaluating CIDP patients and keeping increased intracranial pressure in mind, especially if they already have risk factors.
